# Monascin ameliorate inflammation in the lipopolysaccharide-induced BV-2 microglial cells via suppressing the NF-κB/p65 pathway

**DOI:** 10.22038/ijbms.2020.41045.9702

**Published:** 2020-04

**Authors:** Yong-Xiang Shi, Wei-Shan Chen

**Affiliations:** 1 Department of Orthopedics Surgery, 2nd Affiliated Hospital, School of Medicine, Zhejiang University, 88 Jiefang Road, Hangzhou, 310009, Zhejiang, People’s Republic of China

**Keywords:** BV-2, Inflammatory reaction, Microglia, Monascin, NF-κB/p65 pathway

## Abstract

**Objective(s):**

The pathophysiology of neurodegenerative diseases is complicated, in which inflammatory reactions play a vital role. Microglia cells activation, an essential process of neuroinflammation, can produce neurotoxic molecules and neurotrophic factors, which aggravate inflammation and neuronal injury. Monascin, a major component of red yeast rice, is an azaphilonoid pigment with potential anti-inflammatory effects; however, the effects in central nervous system have not been evaluated. Our goal in this project was to explore the therapeutic effect and the underlying mechanism of Monascin, which may be via anti-inflammatory action.

**Materials and Methods:**

We used lipopolysaccharide to induce BV-2 microglial cells in order to form an inflammation model in vitro. The anti-inflammatory effects of Monascin were measured by enzyme-linked immunosorbent assay (ELISA), real time-polymerase chain reaction (RT-PCR), Western Blot and Immunofluorescent staining.

**Results:**

Our data indicated that inflammatory cytokines including interleukin-1β (IL-1β), IL-6, tumor necrosis factor-alpha (TNF-α) and nitric oxide were suppressed by Monascin treatment. Furthermore, the related pro-inflammatory genes were inhibited consistent with the results of ELISA assay. Western blotting results showed that the phosphorylation of nuclear factor kappa B (NF-κB/p65) was reduced by Monascin treatment may be through suppressing the activation of IκB. Furthermore, immunofluorescence staining showed that the translocation of NF-κB/p65 to the cellular nuclear was blockaded after Monascin treatment.

**Conclusion:**

Taken together, Monascin exerts anti-inflammatory effect and suppressed microglia activation, which suggested its potential therapeutic effect for inflammation-related diseases.

## Introduction

Inflammatory reaction is a common pathological process in numerous neurological diseases including acute brain injury (1), multiple sclerosis (2), Parkinson’s disease and Alzheimer’s disease (3). In the central nervous system (CNS), microglia cells as the major resident immunocompetent cells will be activated firstly to confront pathological changes and regulate neuroinflammatory process, which exerted both neuroprotective and neurotoxic effects in mediating inflammatory responses (3, 4). However, microglia cells were over-activated after various neurological diseases, such as spinal cord injury or cerebral ischemic injury (5). Moreover, activated microglia could release abundant pro-inflammatory factors including interleukin-6 (IL-6), tumor necrosis factor-alpha (TNF-α), IL-1β, and nitric oxide (NO), which will further stimulate the resting microglia to activate and contribute to neuronal death and exacerbate CNS damage (6-9).

In recent *in vitro* studies, it has been reported that nuclear factor kappa B (NF-κB or p65), a downstream of Toll-like receptors signaling pathway, is a family of transcription factors, which is related to macrophages activation and inflammatory mediator release (10). Normally, NF-κB/p65 is integrated by binding to the inhibitor protein IκBα and is present in the cytoplasm in an inactive form (11). 

The upstream regulator of IκBα will be activated when microglia is stimulated by inflammatory factors, then the activated IκBα will be phosphorylated and degraded, which will cause the translocation of NF-kB/p65 to the cellular nucleus and binding to inflammation-related genes leading to improved pro-inflammatory mediators translation (6, 12). Mass of studies have demonstrated that suppressing the NF-κB/p65 signaling pathway results in the amelioration of inflammation-related diseases, such as neuroinflammation (13), experimental colitis (14) and sepsis (15). 

Monascin, a traditional healthy diet that is widely used in Asian, has been reported for its numerous bio-effects including antioxidative, anti-inflammatory, anti-diabetes, and immunomodulatory properties (16-22). Previous studies have indicated that Monascin can decrease the inflammatory reaction via suppressing the release of inflammatory cytokines in IL-1β-induced chondrocyte (23). Moreover, suppression of NF-κB/IκBα pathway by Monascin have been widely shown in different cells, such as monocyte (22), chondrocyte (23), and endothelial cells (24). However, the anti-inflammatory effects and its underlying mechanism of Monascin on lipopolysaccharide (LPS)-induced microglia is unclear.

The potential mechanism in microglia by Monascin treatment may need further evaluation; however, suppression of microglia activated by inflammatory factors is considered a promising therapeutic strategy for neurodegenerative diseases. Thus, our group investigated the anti-inflammatory effect of Monascin and the underlying mechanism *in vitro*, which may be related to NF-κB/p65 signaling pathway.

## Materials and Methods

***Cell culture and treatment***

The microglial cell line (BV-2, CRL-2468) was obtained from the American Type Culture Collection (ATCC, Manassas, USA). BV-2 cells were cultured with complete medium (high glucose DMEM medium added 10% fetal bovine serum) and cultivated at cell incubator. Monascin (CAS No.21516-68-7, purity ≥97 %, Sigma-Aldrich, USA) was prepared as stock solution first, which dissolved in dimethyl sulfoxide (DMSO, Sigma-Aldrich) to form a 100 mM final concentration. LPS (*Escherichia coli* 055:B5, Sigma-Aldrich) was dissolved in saline, and prepared as a stock solution with the final concentration of 5 mg/ml. The stock solution of Monascin was diluted appropriately with cell culture medium (final DMSO concentration ≤ 1%). BV-2 cells were pre-treated with varying dose of Monascin (5, 10, 15, or 20 μM) with or without 1 μg/ml of LPS stimulation for 24 hr.

***Cell viability assay***

BV-2 cells were seeded into a 96-well plate. After 24 hr for adherence, cells were treated with different doses of Monascin (5, 10, 15, or 20 μM) with or without 1 μg/ml of LPS stimulation for 24 hr. Then, 10 μl of CCK-8 (Dojindo, Kumamoto, Japan) solution were added into each well of 96-well plate and cultured in cell incubator for 4 hr. After incubation, spectrophotometer (Bio-Rad, Hercules, USA) was used to detect the OD value at 450 nm, then the cell viability was calculated according to the formula (25): 

Cell survival rate=[(experimental hole absorbance-blank hole absorbance)/(control hole absorbance-blank hole absorbance)]×100%.

***Nitric oxide assay***

Griess reaction was used to measure the Nitrite, which is representative of NO production. Briefly, BV-2 cells were seeded into a 96-well plate. After 24 hr for adherence, cells were treated with different doses of Monascin (5, 10, 15, or 20 μM) with or without 1 μg/ml of LPS stimulation for 24 hr. After incubation, the culture supernatants (100 μl) of each group were added into a 96-well plate, which mixed with 100 μl Griess reagent (Beyotime, Shanghai, China) for 10 min at room temperature in the dark. After incubation, spectrophotometer was used to detect the OD value at 540 nm. 

***ELISA assay***

BV-2 cells were seeded into a 12-well plate. After 24 hr for adherence, cells were treated with different doses of Monascin (5 and 10 μM) with or without 1 μg/ml of LPS stimulation for 24 hr. To detect the anti-inflammatory effect of Monascin, the cell-free supernatants of each well were harvested. The concentrations of IL-1β (MLB00C, sensitivity: 4.8 pg/ml, assay range: 12.5-800 pg/ml), IL-6 (M6000B, sensitivity: 1.8 pg/ml, assay range: 7.8-500 pg/ml) and TNF-α (MTA00B, sensitivity: 7.21 pg/ml, assay range: 10.9-700 pg/ml) were detected by the ELISA kits (R&D Systems, USA) according to the manufacturer’s protocols, and OD values were measured by a spectrophotometer at 450 nm.

***Real-time PCR***

BV-2 cells were re-plated in 6-well plates. After 24 hr for adherence, cells were treated with different doses of Monascin (5 and 10 μM) with or without 1 μg/ml of LPS stimulation for 24 hr. After treatment, TRIzol reagent (Invitrogen) was added into each well to extract the RNA. Then, 1 ul of RNA solution was added into NanoDrop (Thermo Fisher, USA) to detect the concentration and purity of total RNA. 1 μg of total RNA was reverse transcribed into cDNA for the PCR amplification. All cycle threshold values of each gene were harvested and normalized to the β-actin, a housekeeping gene. In order to find the relative difference in each gene, we used the 2^-ΔΔCt ^method. The primers of all genes used for real-time PCR were designed by and Songon Biotech (Shanghai, China) and shown in Table 1. 

***Western blotting***

BV-2 microglia were seeded in a 6-well. After 24 hr for adherence, cells were treated with different doses of Monascin (5 and 10 μM) with or without 1 μg/ml of LPS stimulation for 24 hr. After treatment, 100 μl of RIPA solution (Solarbio, Shanghai, China) containing 1% protease inhibitor (Solarbio) were added to BV-2 cells for 5 min on the ice. Then, the cells were collected by cell scraping and placed in a centrifuge tube followed by 12000 rpm centrifugation at 4 ^°^C for 30 min, the supernatant protein solution was harvested, and protein quantification was performed according to the manufacturer’s protocols of BCA kit (Beyotime, Shanghai, China). The protein was electrophoresed on different concentrations of polyacrylamide gel and then transferred to a polyvinylidene fluoride membrane (Merck, Darmstadt, Germany). Membranes were blocked by 5% skim milk powder for 2 hr and then incubated with primary antibody at 4 ^°^C for 12 hr. Primary antibodies include anti-p-IκBα (1:500, Cell Signaling Technology, rabbit monoclonal antibody, #2859), anti-IκBα (1:500, Cell Signaling Technology, rabbit monoclonal antibody, #4812), anti-p-p65 (1:1000, Abcam, rabbit polyclonal antibody, ab97726), anti-p65 (1:1000, Abcam, rabbit monoclonal antibody, ab32536) and anti-GAPDH (glyceraldehyde-3-phosphate dehydrogenase; 1:500, Cell Signaling Technology, rabbit monoclonal antibody, #5174). Then, membranes were incubated with horseradish peroxidase (HRP)-conjugated secondary antibody (1:1000, Cell Signaling Technology, #7074) for 2 hr in shake cultivation. The membranes were visualized with an electrochemiluminescence plus reagent (Millipore, USA), and images of protein bands were captured on a Chemi DocXRS^+^ Imaging System (Bio-Rad, Hercules, USA). All experiments were repeated three times. The intensity of bands was normalized to those of GAPDH using Image Lab 3.0 software.

***Immunofluorescent staining***

BV-2 cells were seeded into 6-well plates. After 24 hr for adherence, cells were treated with different doses of Monascin (5 and 10 μM) with or without 1 μg/ml of LPS stimulation for 24 hr. After treatment, 1 ml of 4% paraformaldehyde was added into each well for 30 min. Then, 1 ml of 0.5% Triton X-100 was added into each well for 15 min for permeabilization, followed by blocking with 5% bovine serum albumin for 1 hr at room temperature. BV-2 cells were incubated with primary antibodies: anti-p65 (1:400, Abcam, rabbit polyclonal antibody, ab97726) or anti-Iba-1 (1:500, Abcam, rabbit monoclonal antibody, ab178847) at 4 ^°^C for 12 hr. After incubation, Alexa Fluor 488 conjugated secondary antibody (1:800, Abcam, ab150077) was added for 1 hr at 37 ^°^C in the dark. After incubation, cell nucleus was staining with DAPI (Beyotime). The BV-2 cells were visualized by a fluorescence microscope (Leica, Germany). Finally, each well of cells was selected three fields of view randomly and the fluorescence intensity or positive cell number were measured by Image Pro Plus.

***Statistical analysis***

The results were presented as mean±SD. Statistical analyses were performed using SPSS statistical software program 18.0 from three independent experiments. Difference among groups was assessed by the one-way analysis of variance (ANOVA) followed by Tukey test. *P value < 0.05 was considered as statistically significant.

## Results

***The viability of microglia after Monascin treatment***

**Figure 1:**
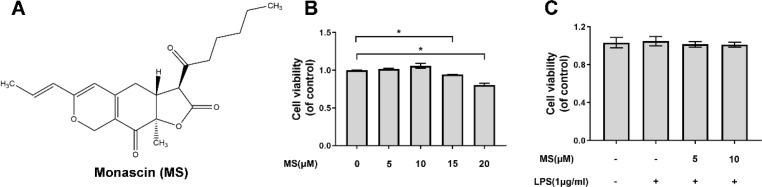
Effects of Monascin on the viability of BV-2 cells

The chemical structure of Monascin was exhibited in Figure 1A. Before investigating the biological function of Monascin on BV-2 microglia, we used CCK-8 method to analyze the potential cytotoxic effects of Monsacin. We found that Monascin did not have cytotoxic effects in microglia between 0-10 μM as shown in Figure 1B. However, microglia after treatment with 15 and 20 μM Monascin showed decreased viability to 94.45% and 80.55%, respectively. Moreover, after stimulation of LPS (1 μg/ml), the viability of microglia treated with 0-10 μM Monascin did not show statistically significant change compared to control group (Figure 1C). Thus, in our experiments, we chose the 10 μM dose of Monascin.

***Monascin inhibits inflammation-related cytokines and genes in LPS-induced microglia***

**Figure 2:**
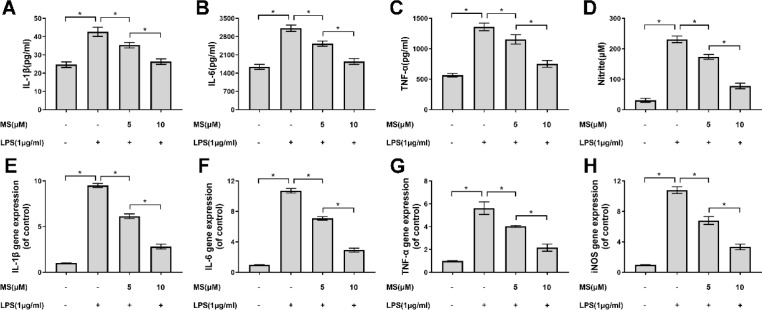
Effects of Monascin on production of pro-inflammatory mediators in lipopolysaccharides (LPS)-induced BV-2 cells

The level of inflammation-related cytokines could reflect the extent of inflammatory response. In our experiments, we detected the content of IL-1β, IL-6, TNF-α and NO after LPS stimulation in microglia. Our results showed that in LPS-induced group, the concentration of pro-inflammatory cytokines including IL-1β, IL-6 and TNF-α remarkably increased, which significantly reversed by Monascin treatment at doses of 5 and 10 μM (Figure 2A-2C). Meanwhile, we investigated the production of NO after Monnasin treatment, which was synthesized by inducible nitric oxide synthase (iNOS). As shown in Figure 2D, LPS induced a notable release of NO. However, pretreatment with Monascin significantly decreased LPS-induced NO production. Furthermore, we used qPCR to determine the level of inflammatory-related mRNA. Similarly, IL-1β (Figure 2E), IL-6 (Figure 2F), TNF-α (Figure 2G) and iNOS (Figure 2H) mRNAs were significantly enhanced following LPS stimulation and remarkably reduced by Monascin pretreatment. The results indicate that Monascin negatively controls the pro-inflammatory mediator production of IL-1β, IL-6, TNF-α and NO. Moreover, it could downregulate IL-1β, IL-6, TNF-α and iNOS mRNAs at the transcriptional level.

***Monascin inhibits LPS-induced microglia activation***

**Figure 3:**
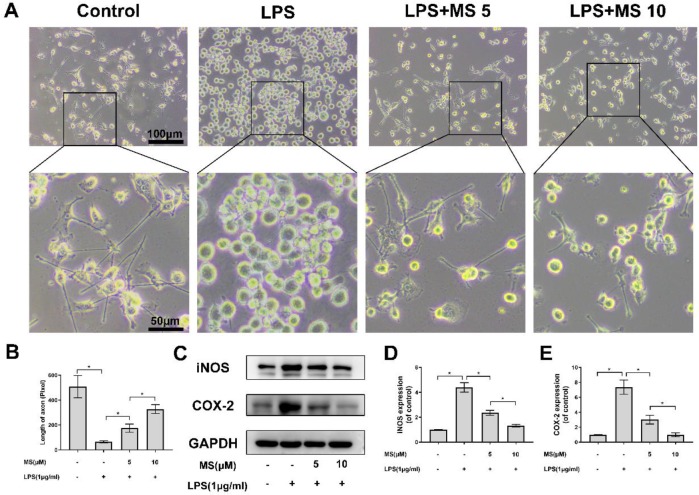
Monascin suppressed lipopolysaccharides (LPS)-induced microglial cells activation

Microglia can change their morphology to accommodate various environments. For instance, microglia stimulated by LPS, IFN-γ and β-amyloid would retract the axon, which show branching morphology in normal conditions. As shown in Figure 3A, microglia shifted to an amoeboid shape by LPS stimulation, indicating that microglia are activated; however, microglia develop branching morphology after Monascin treatment. In Figure 3B, the length analysis results showed that the axonal length of microglia decreased around at 65.25 pixels 24 hr after LPS stimulation compared to control group (509.5 pixels) and restored to 175.5 and 328.5 pixels by 5 and 10 μM of Monascin treatment, respectively. Furthermore, iNOS and cyclooxygenase-2 (COX-2) that were markers of activated microglia were detected by western blot, suggesting that the expression of iNOS and COX-2 were remarkably suppressed by Monascin pretreatment (Figure 3C-3E). Overall, these data could show that microglia could be activated by LPS simulation, which complied with morphological transformation. Meanwhile, pretreatment of Monascin could inhibit those effects of LPS.

***Monascin inhibits the Iba-1 expression of LPS-induced microglia***

**Figure 4.**
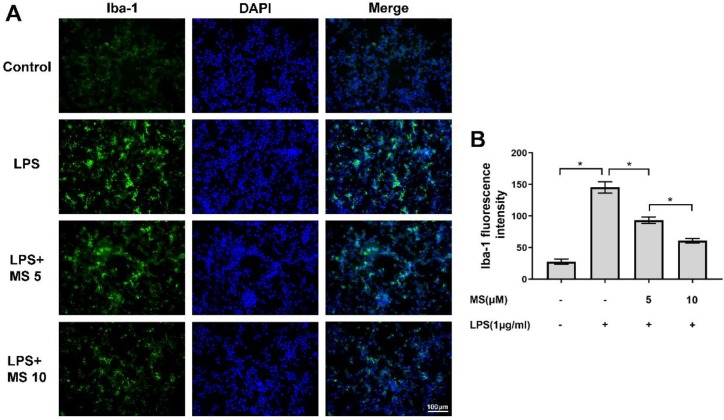
Monascin suppresses the Iba-1 expression of microglia

Iba-1 is a main marker of microglia, which is activated by LPS. In our study, we detected the Iba-1 marker by immunofluorescence to assess the anti-inflammatory effect of Monascin. As shown in Figure 4A and 4B, Iba-1 fluorescence intensity was notably increased after LPS stimulation for 24 hr. Interestingly, it could be reversed by Monascin pretreatment (Figure 4A and 4B). These data demonstrated that Monascin could suppress microglia activation through suppressing the expression of Iba-1.

***Monascin inhibits NF-κB/p65 signal pathway in LPS-induced microglia***

**Figure 5:**
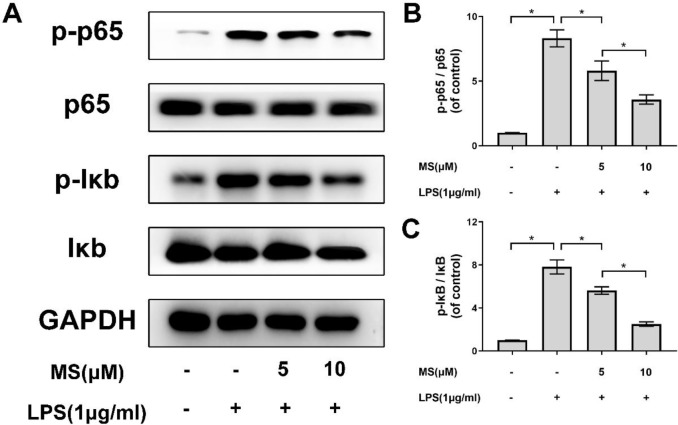
Monascin regulates activation of the nuclear factor kappa B (NF-κB/p65) pathway

Our group found that NF-κB or p65 signal pathway plays a vital role in the underlying mechanism of anti-inflammatory effects of Monascin. The western blot showed that the expression of NF-κB/p65 pathway-related protein, the phosphorylation of NF-κB/p65 and IκBα levels were significantly enhanced by LPS stimulation compared to control group, and markedly downregulated after Monascin treatment compared to that of LPS-stimulated group (Figure 5A-5C). Moreover, Monascin remarkably reversed the activation of NF-κB/p65 signal pathway compared to the LPS group in a dose-dependent manner. Furthermore, immunofluorescent staining was implemented to examine the nuclear translocation of NF-κB/p65.

**Figure 6:**
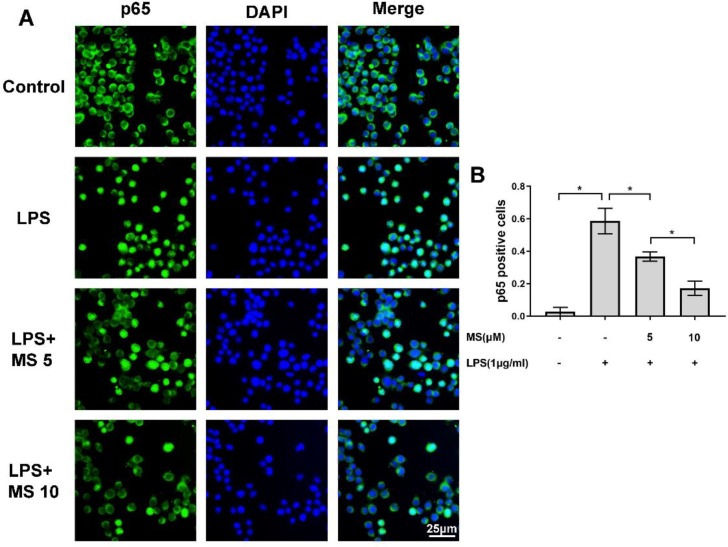
Monascin blocked the transfer of nuclear factor kappa B (NF-κB/p65)

The immunofluorescent staining results indicated that most of the NF-κB/p65 proteins were settled at cytoplasm in resting microglia. On the contrary, when microglia induced by LPS, NF-κB/p65 proteins will translocate to the nucleus. However, this phenomenon of NF-κB/p65 protein nuclear translocation was blocked by Monascin pretreatment (Figure 6A and 6B). In summary, these results indicate that NF-κB/p65 signal pathway plays an essential role in microglia activation, which could be suppressed by Monascin.

## Discussion

Monascus-fermented rice is a popular food that has been used for more than 1,000 years in many Asian countries and is considered as a traditional healthy diet. Monascin, an azaphilonoid pigment, is the main components of Monascus-fermented rice (21). Numerous studies have reported that Monascin could exert many bioactive effects including anti-inflammation, anti-oxidation and anti-diabetic (19, 20, 22). However, its protectiveness in neurodegenerative diseases remains unknown. 

In this study, we indicated the inhibition of inflammation and elucidated the molecular mechanism by which Monascin is regulated in microglia.

Nitric oxide synthase (NOS) is an enzyme present in endothelial cells, macrophages, neurophagocytes, and nerve cells. iNOS is expressed after injury.NO is also derived from iNOS, and it has been shown that neuronal NOS has a neurotoxic effect (26-28). In our present study, Monascin inhibited the NO synthesis by expression of iNOS mRNA. Moreover, inflammatory response is a positive feedback cascade, and the expression of iNOS could be remarkably enhanced by the other secretory pro-inflammatory cytokines (29-31). Previous studies have indicated that Monascin could inhibit inflammation-related cytokines expression in IL-1β-induced chondrocytes (23). These results are consistent with our present research that Monascin inhibits the production of TNF-α, IL-1β, and IL-6, which significantly increased by LPS stimulation. Meanwhile, Monascin downregulates the relevant mRNA expression. In CNS, inflammation along with releasing TNF-α, IL-1β and chemokines, aggravates inflammatory reaction and exacerbates the damage of neurons (32, 33). Similarly, COX-2, an inflammatory enzyme, takes parts in the process of inflammatory response and microglia activation. It could be considered as another essential enzyme in inflammation, which could be generated by microglia and astrocytes (34, 35). In our present study, we demonstrated that Monascin had no cytotoxic effects on microglia viability at concentrate of 5 and 10 μM, which were used in our experiments. A mass of studies has reported that neurodegenerative diseases may relate to microglia activation, though the underlying mechanism of microglia activation or though the neurological process that are not clear (36, 37). Our data suggest that Monascin may inhibit pro-inflammatory mediators including TNF-α, IL-1β, IL-6, COX-2 and iNOS at the gene and protein level. Thus, Monascin is identified to have anti-inflammatory effects on microglia.

NF-κB/p65 plays a major role in starting of inflammatory reactions. Moreover, it could regulate the transcription of inflammatory mediators including iNOS and COX-2 (38, 39). A complex formed by NF-κB/p65 and IκB-α settles in the cytoplasm in the resting cells. When the NF-κB/p65 signal pathway is activated, the extracellular stimulating factors such as LPS and IL-1β, as well as the complex formed by IκB-α and NF-κB/p65 will be separated to monomer and will subsequently phosphorylate each other. Finally, the phosphorylated NF-κB/p65 will translocate to the nuclear region. In order to further investigate the underlying mechanism of the anti-inflammatory response of Monascin, we used western blot and immunofluorescence assay. The western blot assay indicated that the NF-κB/p65 signal pathway was activated after LPS stimulation, which could be reversed by Monascin pretreatment. Furthermore, immunofluorescence assay showed that NF-κB/p65 translocate to the nuclear remarkably increased by LPS stimulation and reversed by Monascin pretreatment. 

**Table 1:** Primers used for real-time PCR analysis

Genes	Forward primers	Reverse primers
IL-6	TCCATCCAGTTGCCTTCTTG	ATTGCCATTGCACAACTCTTTT
IL-1β iNOS TNF-α	TGGACCTTCCAGGATGAGGACA TCCCAGCCTGCCCCTTCAAT CTCAAGCCCTGGTATGAGCC	GTTCATCTCGGAGCCTGTAGTG CGGATCTCTCTCCTCCTGGG GGCTGGGTAGAGAACGGATG

IL-1β: interleukin 1 beta, TNF-α: Tumor necrosis factor alpha, iNOS: Inducible nitric oxide synthase

(A) Chemical structure of Monascin. (B, C) BV-2 cells were treated with Monascin (0-20 μM) in the absence or presence of lipopolysaccharides (LPS) for 24 hr, and the cell viability was measured by CCK8 assay. The values presented are the means±SD. *P<0.05 vs. control group, n=3

BV-2 cells were treated with 5 or 10 μM Monascin with or without 1 μg/ml of LPS for 24 hr. The levels of A interleukin 1 beta (IL-1β), B IL-6, C tumor necrosis factor alpha (TNF-α), and D nitric oxide (NO) and E IL-1β, F IL-6, G TNF-α, and H inducible nitric oxide synthase (iNOS) mRNA were measured. Results are expressed as the mean of three independent experiments. *P<0.05 vs. indicated group

(A, B) The morphological results of BV-2 cells treated with Monascin (5 or 10 μM) in the absence or presence of LPS for 24 hr. Morphological changes were quantified by measuring the length of axon. (C) Western blot analysis of inducible nitric oxide synthase (iNOS) and cyclooxygenase-2 (COX-2). Monascin significantly reversed the inflammation enzyme synthesis in microglia after LPS stimulation in a dose-dependent manner. The densitometric analysis of all Western blot bands was normalized to glyceraldehyde-3-phosphate dehydrogenase (GAPDH). The values presented are the means±SD. *P<0.05 vs. indicated group, n=3

(A, B) Immunofluorescence staining results of Iba-1 by Monascin and/or lipopolysaccharides (LPS) treatment in microglial cells. BV-2 cells were treated with 5 or 10 μM Monascin with or without 1 μg/ml LPS for 24 hr. Monascin markedly inhibited Iba-1 expression, which had been increased by LPS. The values presented are the means ± SD. *P<0.05 vs. indicated group, n=3

(A, B, C) Western blot analysis of p-IκB and p-p65 at different doses of Monascin treatment. Lipopolysaccharides (LPS) evidently activated NF-κB pathway, which was reversed by Monascin. The densitometric analysis of all Western blot bands was normalized to glyceraldehyde-3-phosphate dehydrogenase (GAPDH). The values presented are the means±SD. *P<0.05 vs. indicated group, n=3

(A) Immunofluorescence staining results of p65 in Monascin and/or lipopolysaccharides (LPS)-treated microglial cells. Monascin ameliorated NF-κB/p65 translocation into the nucleus in microglial cells. The values presented are the means±SD. *P<0.05 vs. indicated group, n=3

## Conclusion

Taken together, the present *in vitro* study indicates that Monascin may suppress the NF-κB/p65 signal pathway through inhibiting IκB degradation, which relates to the pro-inflammatory factors release and microglia activation. Moreover, we clarified the underlying anti-inflammation mechanism of Monascin in LPS-induced microglia and provided a theoretical basis for its success in the clinical treatment of neurodegenerative diseases.
